# Peritoneal regression grading score (PRGS): first evidence for independent predictive and prognostic significance

**DOI:** 10.1515/pp-2023-0014

**Published:** 2023-05-18

**Authors:** Janina Baake, Giorgi Nadiradze, Rami Archid, Alfred Königsrainer, Hans Bösmüller, Marc Reymond, Wiebke Solass

**Affiliations:** National Center for Pleura and Peritoneum, Comprehensive Cancer Center South-Western Germany, Tübingen-Stuttgart, Germany; Department of General and Transplant Surgery, Eberhard-Karls-University Tübingen, Tübingen, Germany; Institute of Pathology, Eberhard-Karls-University Tübingen, Tübingen, Germany; Institute of Tissue Medicine and Pathology, University Bern, Bern, Switzerland

**Keywords:** antineoplastic agents, metastasis, neoplasm, pathology

## Abstract

**Objectives:**

The peritoneal regression grading score (PRGS) is a four-tied pathologic score measuring tumor regression in biopsies from patients with peritoneal metastasis (PM) receiving chemotherapy.

**Methods:**

This retrospective analysis of a prospective registry (NCT03210298) analyses 97 patients with isolated PM under palliative chemotherapy. We examined the predictive value of the initial PRGS for overall survival (OS) and the prognostic value of PRGS in repeated peritoneal biopsies.

**Results:**

The 36 (37.1 %) patients with an initial mean PRGS≤2 had a longer median OS (12.1 months, CI 95 % 7.8–16.4) vs. 8.0 months (CI 95 % 5.1–10.8 months) in 61 (62.9 %) patients with PRGS≥3 (p=0.02) After stratification, the initial PRGS was an independent predictor of OS (Cox-regression, p<0.05). Out of 62 patients receiving≥two chemotherapy cycles, 42 (67.7 %) had a histological response (defined as a lower or stable mean PRGS in successive therapy cycles), and 20 (32.3 %) progressed (defined as an increasing mean PRGS). PRGS response was associated with a longer median OS (14.6 months, CI 5–95 % 6.0–23.2) vs. 6.9 (CI 5–95 % 0.0–15.9) months. PRGS response was prognostic in the univariate analysis (p=0.017). Thus, PRGS had both a predictive and prognostic significance in patients with isolated PM receiving palliative chemotherapy in this patient cohort.

**Conclusions:**

This is the first evidence for the independent predictive and prognostic significance of PRGS in PM. These encouraging results need validation in an adequately powered, prospective study.

## Introduction

Therapy response assessment in patients with peritoneal metastasis (PM) is a particular challenge in modern oncology. RECIST 1.1, as assessed by CT-scan, is the reference system for evaluating tumor response in oncology studies [1]. However, in patients with peritoneal metastasis, the accuracy of current imaging systems is limited. The healthy peritoneum is a single-layered membrane [2] that CT-scan can barely visualize. PM is often a small-volumetric disease with a diameter of lesions at the low detection limit of cross-sectional imaging methods such as CT [3]. Moreover, the patient’s breathing and bowel motility can cause a loss of image sharpness. Unfortunately, in the absence of target lesions, CT-based determination of progression-free survival or time-to-progression is impossible in patients with PM. Therefore, laparoscopy is increasingly used to determine the response of PM to chemotherapy [4, 5] and to indicate cytoreductive surgery,

Laparoscopy allows macroscopic staging of the extent of peritoneal disease and biopsies from tumor-suspect areas. A prognostic significance of tumor regression grading in peritoneal metastasis has already been documented in the neoadjuvant setting in colorectal and ovarian cancer [6]. In the palliative setting, a group of pathologists in Europe proposed in 2016 a novel regression grading for therapy response assessment in PM, the peritoneal regression grading score (PRGS) [7]. PRGS is a four-tied grading system considering several specific characteristics of PM, in particular their frequent mucinous character. Peritoneal biopsies are taken from all four abdominal quadrants to consider tumor heterogeneity. PRGS is determined as follows: Grade 1: complete response (absence of tumor cells), Grade 2: major response (major regression features, few residual tumor cells), Grade 3: minor response (some regressive features but predominance of residual tumor cells), Grade 4: no response (tumor cells without any regressive features). Acellular mucin and infarct-like necrosis are regarded as regression features. Then, the mean PRGS is calculated from all biopsies available.

Since 2016, PRGS is diffusing rapidly into clinical practice worldwide [8], [9], [10], [11], [12], [13], [14], [15], [16], [17], [18], [19], [20], [21], [22], [23], [24], [25], [26], [27], [28] underscoring the high need for a uniform histological therapy response score in PM. In the meantime, extensive methodological and validation work has been done on PRGS. In a multicentric study on 331 peritoneal biopsies, the interobserver variability for PRGS of each biopsy and the mean PRGS per biopsy set was moderate to good/substantial. The intra-observer variability for PRGS of each biopsy and the mean PRGS per biopsy set was good to excellent/almost perfect [29]. The minimal number of biopsies required for determining PRGS with an accuracy superior to 80 % was found to be three, and grading “PRGS_x_” was recommended for the cases with two or fewer biopsies [30]. Finally, the value of additional staining in biopsies with no tumor cells visible was evaluated: Further staining reduced the instances of unclear PRGS to zero and changed the therapy response assessment in 21 % of patients [31].

Many patients selected for locoregional therapy with cytoreductive surgery, HIPEC and/or PIPAC receive preoperative systemic chemotherapy. In surgical, oncology, when it comes to the efficacy of metastasectomy, “Biology is King, and selection of cases is Queen” [32]. Intuitively, patients with chemoresistant tumors (highly vital tumors, PRGS 3 and 4) are expected to benefit less from aggressive surgery than patients with chemosensitive tumors. However, at the present time, conclusive evidence on the predictive and prognostic significance of PRGS in clinical practice is still largely missing. This study delivers solid evidence that PRGS allows to stratify patients with PM based on long-term outcomes, improving patient selection for surgical, potentially curative therapy, vs. medical, palliative therapy.

## Patients and methods

### Study design

This is a registry study on a cohort of consecutive patients with histologically documented peritoneal metastasis first treated between 1.7.2016 and 1.5.2019 in a tertiary care center specialized in multimodal therapy or peritoneal metastasis. Data were entered prospectively into a patient registry, and the analysis was retrospective. No primary/secondary endpoint was defined in advance.

### Ethical and regulatory framework

Clinical, pathology and follow-up data were entered into the international PIPAC patient registry (www.clinicaltrials.gov, NCT03210298). This registry was approved by the Ethics Committee, Ruhr-University Bochum, on January 11th, 2016 under the reference 15–5,280). Each patient gave their written informed consent for data storage management and analysis. The retrospective analysis was authorized by the Ethics Committee, University of Tübingen, on 18.3.2021, under the reference 073/2019BO.

### Therapy

All patients received intraperitoneal chemotherapy as pressurized intraperitonel aerosol chemotherapy (PIPAC) with oxaliplatin 92 mg/m^2^ BSA or with a combination of cisplatin 7.5 mg/m^2^ BSA and doxorubicin 1.5 mg/m^2^ BSA. A majority of patients (64.9 %) received combined systemic chemotherapy and PIPAC. A total of 244 PIPAC procedures were performed on 97 patients. The mean number of PIPACs administered was 2.5 (minimum one, maximum seven). Sixty-two patients had at least 2 PIPAC. PIPAC cycles were repeated at 6-week intervals. In the case of major or complete histological regression, the time interval was extended to 3 months. In the cases of combined treatment (systemic and intraperitoneal chemotherapy), a minimum delay of two weeks (bevacizumab: 4 weeks) was observed between the last application of systemic chemotherapy and PIPAC.

### Peritoneal biopsy specimens

Millimetric biopsies were taken with a 5-mm endoscopic instrument (ClickLine Biopsy forceps, Karl Storz, Tuttlingen, Germany) from macroscopically tumor suspect areas in all four abdominal quadrants. Peritoneal biopsies were fixed in 10 % buffered formalin for 24–48 h. Then, samples were embedded in paraffin using a controlled temperature. Two series of three 2.5 µm thick step sections from each biopsy were stained with hematoxylin and eosin (H&E).

### Determination of the peritoneal regression grading score (PRGS)

Histopathological tumor regression was graded according to the 4-tied peritoneal regression grading score (PRGS), as described previously [31]. Shortly, PRGS 1 equals complete histological response and is defined as the absence of residual cancer and a large amount of regressive fibrosis (and/or acellular mucin pools and/or infarct-like necrosis). PRGS 2 is defined as a specimen where regressive changes are predominant over cancer cells. PRGS 3 is a specimen where regressive changes are present but where cancer cells are dominant over fibrosis. PRGS 4 is a specimen without histological features of regression. Infarct- or mucin-like necrosis is regarded as indicative of tumor regression. In contrast, unspecific tumor necrosis is indicative of a lack of response in the respective area, as described for colorectal liver metastases [33]. Various independent senior pathologists assessed PRGS by grading tumor biopsies taken during each laparoscopy, and no blinding was applied. The mean PRGS was determined in each patient, thus taking into account all information from available biopsies. PRGS was used for all tumor histologies.

### Follow-up

The closing date was Feb 22nd, 2020, and the mean follow-up was 32.1 months (min 281 days, max 1,417 days). Patients or their general practitioners were contacted by phone or email.

### Statistical analysis

Values are given as means or medians, where appropriate. Comparisons of clinicopathologic features at diagnosis and treatment details were analyzed using the Pearson 2 exact test and Kruskal-Wallis test, as appropriate. Overall survival was defined as the time elapsed between the first intraperitoneal (PIPAC) chemotherapy cycle and death or date of last censored. Overall survival was estimated by the Kaplan-Meier method and compared by the log-rank test. We performed a multivariable logistic regression model (Cox) with survival as the dependent variable and PCI, Karnofsky index, PRGS, timepoint of peritoneal metastasis (synchronous vs. metachronous), number of previous chemotherapy lines, and presence of ascites (yes/no) as independent variables. All p-values are two-tailed, and a p-value of less than 0.05 was considered statistically significant. We used SPSS 24 for Windows (SPSS Inc., Chicago, IL, USA) for statistical analysis and SigmaPlot 13 (Systat Software Inc., San José, CA, USA) for creating graphs.

## Results

A total of 97 consecutive patients with histologically proven, isolated peritoneal metastasis were available for analysis. The patients’ characteristics are summarized in Table 1. Primary tumors were gastric cancer (n=33), hepatobiliary/pancreatic cancer (n=20), colorectal cancer (n=18), ovarian cancer (n=8), appendiceal cancer (n=7), pseudomyxoma peritonei (n=2) and other tumors (n=9, cancer of unknown origin, malignant peritoneal mesothelioma, yolk sac tumor, breast, and prostate cancer). Most patients (n=86) received palliative systemic chemotherapy before the first PRGS determination. The minority (n=11) were chemo-naïve at this point.

**Table 1:** Patient characteristics.

Variable	Value
Number of patients	97
Sex (M:F)	44:53
Age, years (±SD)	55.8 ± 11.8
Organ of origin Gastric Colorectal/appendiceal Hepatobiliary-pancreatic Ovarian PMP Others: CUP, mesothelioma, yolk sac, prostate	33 (34.0 %) 25 (25.8 %) 20 (20.6 %) 8 (8.2 %) 2 (2.0 %) 9 (9.4 %)
Peritoneal metastasis Synchronous Metachronous	56 (57.7 %) 41 (42.3 %)
Extraperitoneal metastasis	9 (9.3 %)
Peritoneal cancer index (PCI), mean ± SD	20.0 ± 12.5
Karnofsky index before first PIPAC, mean ± SD	82.6 ± 13.3
Ascites ≥300 mL	21 (21.6 %)
Previous systemic chemotherapy None ≥1 line	11 (11.3 %) 86 (88.7 %)
Therapy PIPAC Systemic chemotherapy	97 (100 %) 63 (64.9 %)

At first biopsy, the mean PRGS (CI 5–95 %) was higher in chemo-naive patients (2.24; 1.68–2.80) in 14 vs. 1.95 (1.76–2.13) in pre-treated patients, which probably reflects the therapy effect. However, this difference did not reach statistical significance, p=0.24. The proportion of PRGS 1 (no tumor cells detected in multiple peritoneal biopsies) and PRGS 2 (high-grade regression) at initial assessment was higher in HBP and gastric cancer than in other histologies (Table 2). This difference is due to the desmoplastic nature of biliopancreatic cancer [34, 35] and (diffuse) gastric cancer [36], with markedly increased cancer-associated fibroblasts and collagen deposition.

**Table 2:** PRGS at initial assessment, depending on the organ of origin.

	Complete or high-grade regression, PRGS 1 and 2	Vital tumor or minor regression, PRGS 3 and 4
Gastric cancer, n=33	27 (81.8 %)	6 (8.2 %)
HBP cancer, n=20	15 (75.0 %)	5 (25.0 %)
Other primaries, n=44	14 (31.8 %)	30 (68.1 %)
All tumors, n=97	56 (57.7 %)	41 (42.3 %)

The proportion of PRGS 1 (no tumor cells detected in multiple peritoneal biopsies) and PRGS 2 (high-grade regression) at initial assessment was significantly higher in HBP and gastric cancer (mean PRGS 1.57, CI 5–95 % 1.42–1.72) than in other histologies (mean PRGS 2.48, CI 5–95 % 2.22–2.74, p<0.001).

### Predictive value of PRGS for OS

At the end of the follow-up period, 71/97 patients had passed away, and 26 were still alive. For all organs of origin together, the median overall survival (OS) from the first PIPAC was 9.9 months (95 % CI: 6.4–13.4 months). From the time point of diagnosis of PM, the OS was 17.9 months (95 % CI: 14.2–21.7 months).

**Figure 1:**
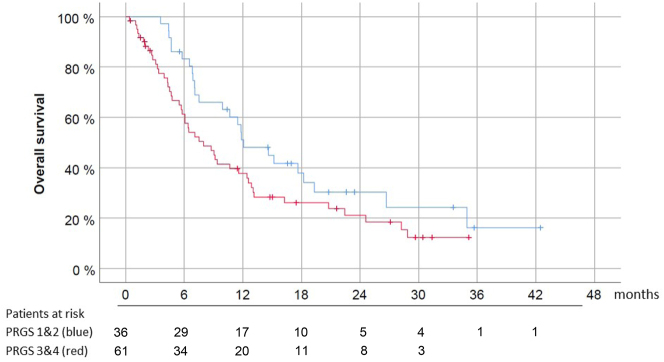
Predictive value of PRGS in 97 patients with isolated peritoneal metastasis. The origin for overall survival. PRGS was grouped into two categories: 1) 36 patients with initial mean PRGS ≤2 with no tumor cell or high-grade regression, and 2) 61 patients with initial mean PRGS >2 with vital tumor or low-grade regression. Most patients (88.7 %) received previous systemic chemotherapy. In the multivariate analysis (Cox-regression model), PRGS (p<0.05) and organ of origin (p<0.001) are independent predictors of survival in this patient population.

The 36 (37.1 %) patients with an initial mean PRGS ≤2 had a significantly longer median OS (12.1 months, CI 5–95 % 7.8–16.4) vs. 8.0 months (CI 5–95 % 5.1–10.8 months) in 61 (62.9 %) patients with mean PRGS >2 (Figure 1).

**Figure 2:**
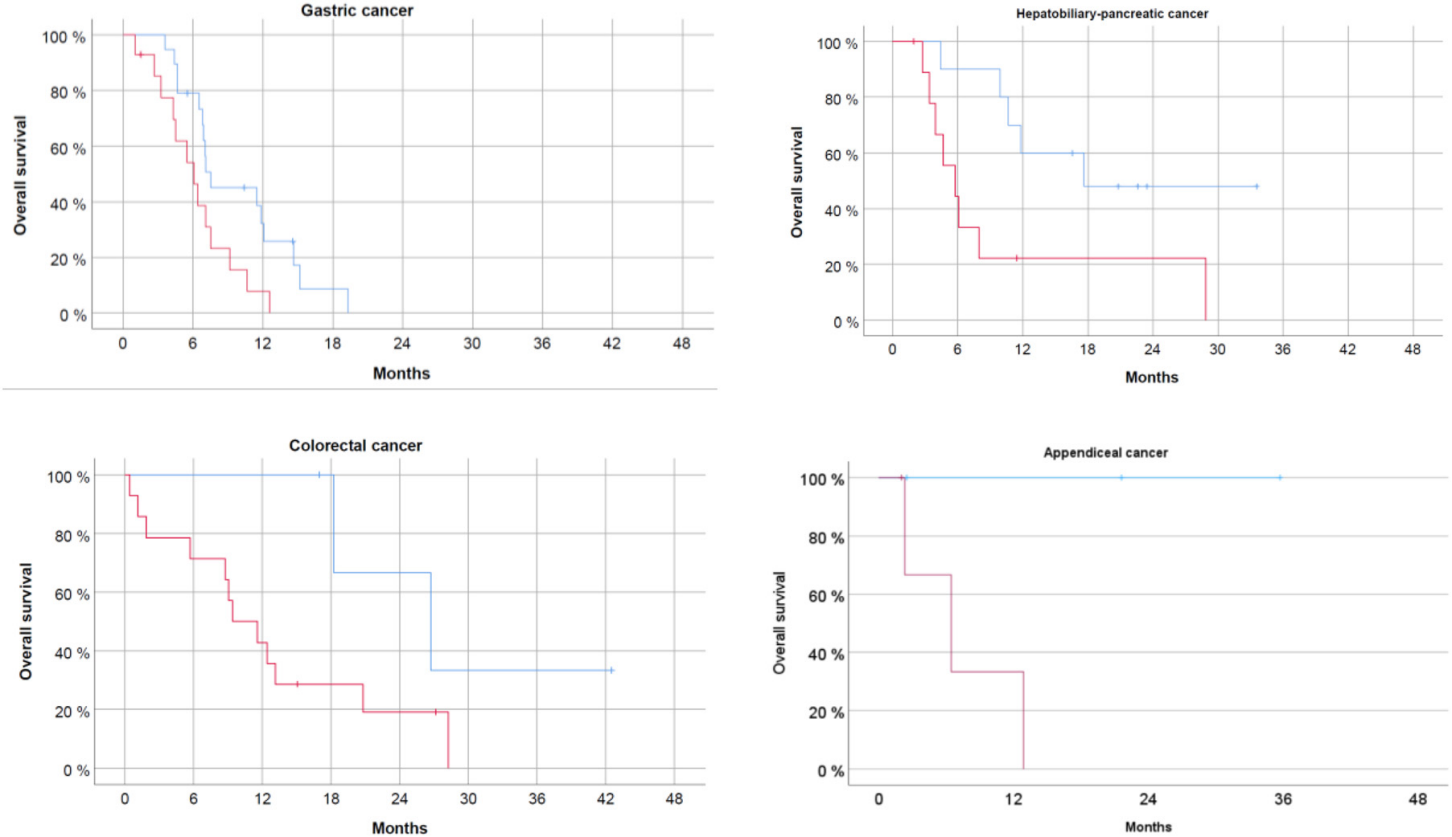
PRGS predicts overall survival of patients with peritoneal metastasis from different primaries. For patients with gastric cancer, colorectal, hepatobiliary-pancreatic and appendiceal cancer, PRGS is a predictor of survival. Patients with a favorable histological score (PRGS ≤2) survived longer than patients with a PRGS >2. Due to the small sample size, the differences do not reach statistical significance in the subgroups.

A multivariate analysis (Cox regression model) was then performed, including established prognostic factors in PM patients: general condition measured with the Karnofsky index, the extent of peritoneal spreading measured with the Peritoneal Cancer Index (PCI), the presence of ascites >300 mL and the organ of origin. Together with the organ of origin (p<0.001), PRGS remained an independent predictor of OS (p<0.05). Then, we calculated the survival curves for each organ of origin with >20 patients included (PM of gastric, colorectal, and hepatobiliary-pancreatic origin). Results are shown in Figure 2.

### Prognostic value of histological tumor regression for overall survival

**Figure 3:**
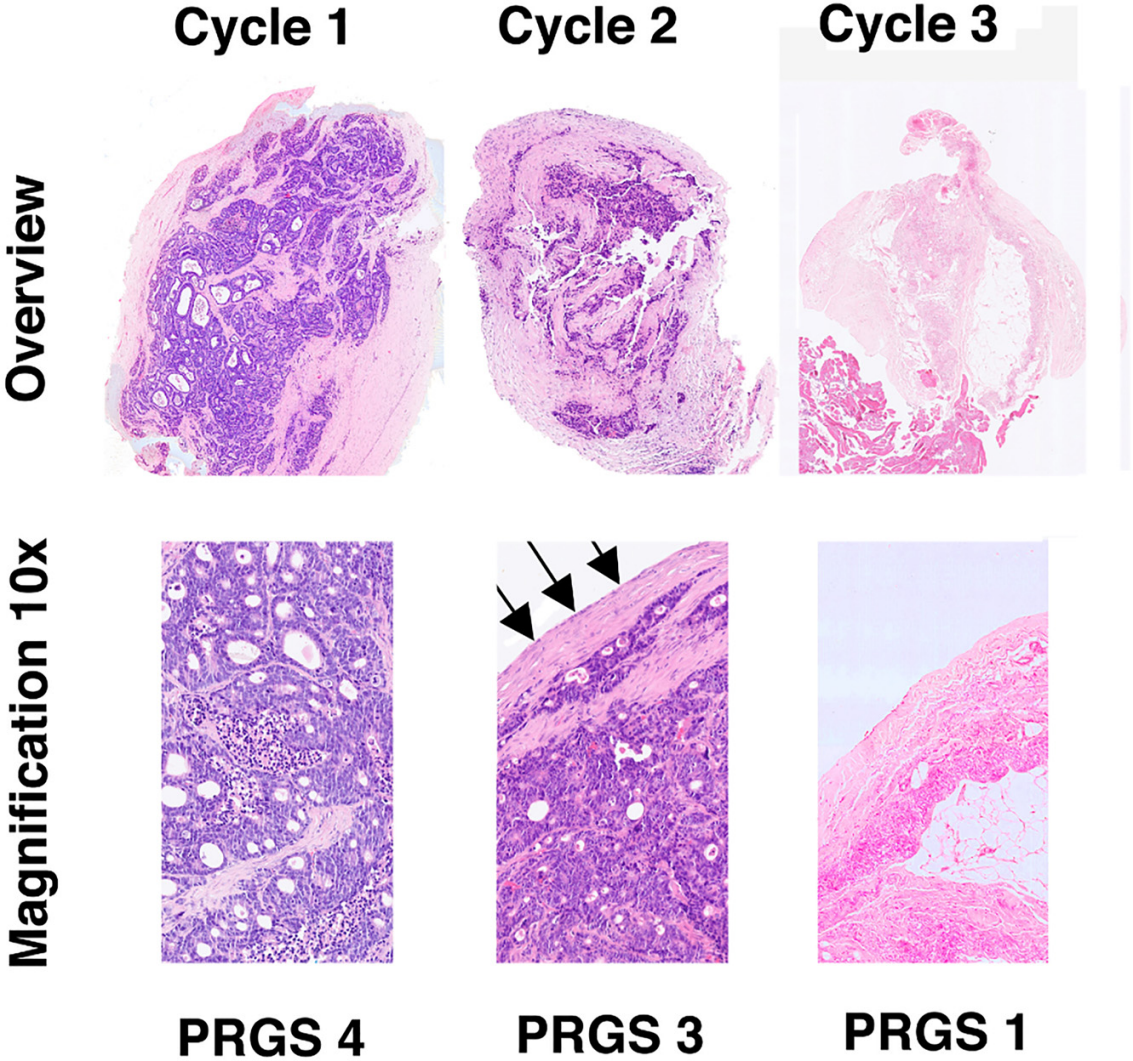
Example of histological regression under palliative chemotherapy. Repeated biopsies and determination of the peritoneal regression grading score (PRGS) in a patient with isolated peritoneal metastasis from the colorectal origin with a progressive disease according to RECIST 1.1 under palliative chemotherapy (6 cycles FOLFOX). Left panel, cycle 1: vital tumor with no sign of regression after intravenous chemotherapy, PRGS 4. Middle panel, cycle 2: minor regression after intensified systemic and intraperitoneal chemotherapy; arrows: the direction of application of PIPAC with typical superficial sclerosis of the tumor node, PRGS 3. Right panel, cycle 3: complete histological regression with no vital tumor cells, PRGS 1. Hematoxyline-eosin (HE) staining.

In 42/62 patients (67.7 %) who received at least two cycles of PIPAC, repeated peritoneal biopsies unraveled an improved or stable PRGS. According to the histological response criteria defined above, this objective evidence of tumor regression or stable disease was considered a therapeutic response. A typical example of such a PRGS regression under intensified palliative chemotherapy is shown in Figure 3.

**Figure 4:**
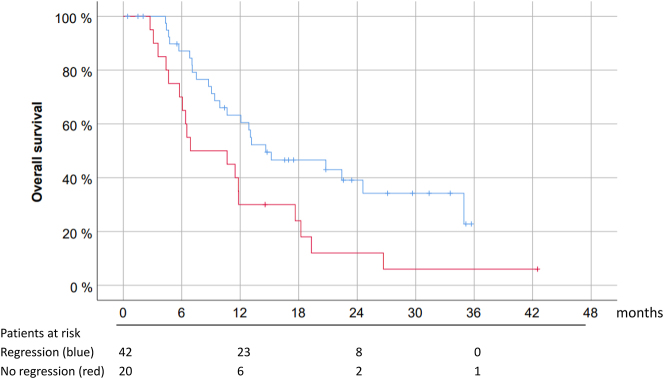
Histological therapy response (assessed as changes in the mean PRGS) is an independent predictor of survival in patients with isolated peritoneal metastasis. Prognostic value of PRGS for overall survival in 62 patients with PM from various primaries with at least 2 consecutive histological assessments in the course of palliative chemotherapy. Patients were grouped into two categories: 1) 42 patients with a histological response (the mean PRGS remained stable or decreased under therapy, blue line), and 2) 20 patients with a histological progression (the mean PRGS increased under therapy, red line). After stratification for organ of origin, the mean PRGS was an independent predictor of survival in this patient population (p<0.02).

Out of the patients who received repeated intraperitoneal chemotherapy, 14/62 patients (22.6 %) had a complete histological regression (PRGS=1) in all peritoneal biopsies with no visible tumor cells. In the remaining 20 patients, PRGS deteriorated under therapy. As shown in Figure 4, patients with a PRGS regression (or stable) have better survival (median 14.6 months, CI 5–95 % 6.0–23.2 months) than patients with a progression (median 6.89 months, CI 5–95 % 0 – 15.9 months), and this difference was significant in the univariate analysis p<0.05. However, in this relatively small patient cohort, PRGS regression did not remain predictive for OS in the multivariate analysis.

**Figure 5:**
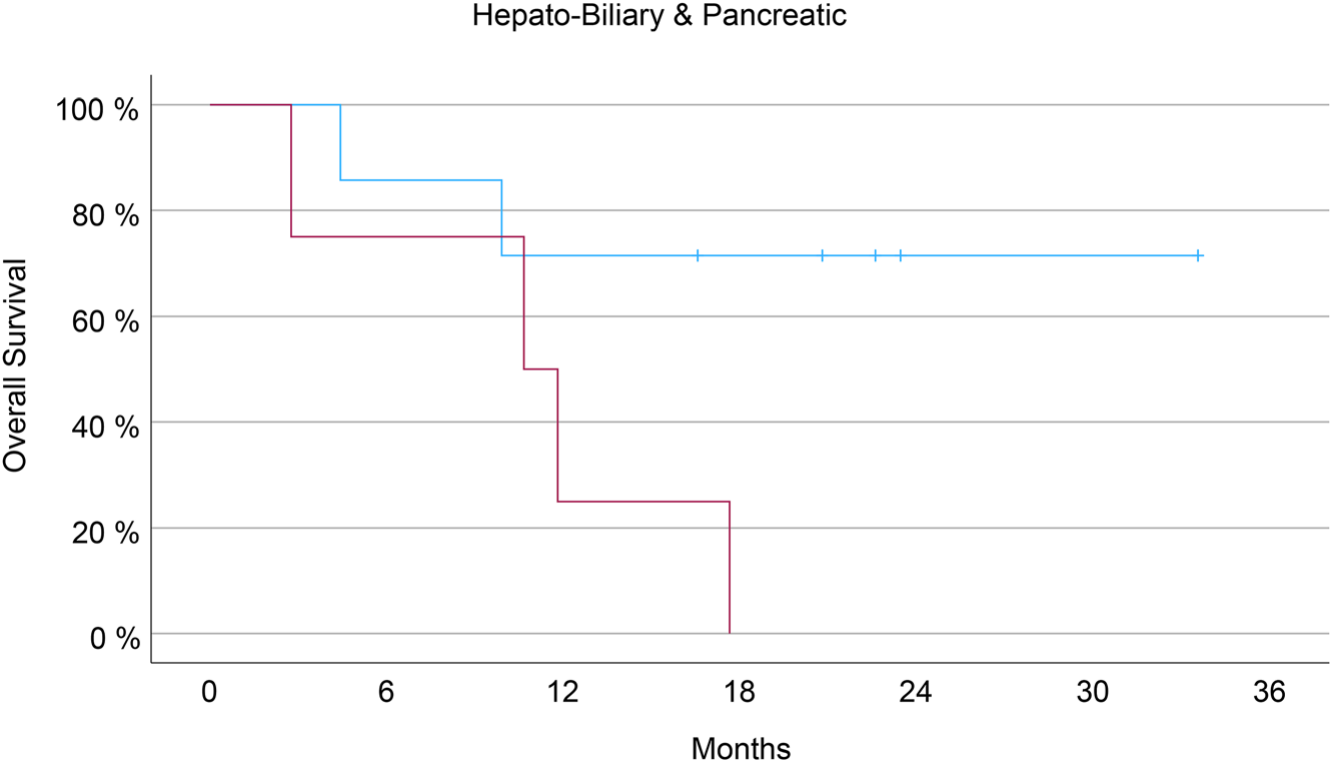
Survival of patients with isolated peritoneal metastasis of hepatobiliary-pancreatic (HBP) origin under palliative chemotherapy. All patients with PRGS progression (red curve) died within 18 months after intervention, which was expected in patients with peritoneal metastasis from HBP cancer. However, there are long-time survivors in the patients with an increase in the mean PRGS, reflecting an objective histological response to chemotherapy. The difference in overall survival between subgroups reaches statistical significance (Kaplan-Meyer, log-rank test, p<0.05).

As shown in Figure 5, the survival difference between responders and non-responders was particularly striking in the 20 patients with HBP tumors. In this group, the PRGS sharply distinguished between the two groups with different outcomes (p=0.06).

## Discussion

Histological regression scores have already demonstrated their clinical utility in liver metastasis of colorectal origin, in the neoadjuvant setting, such scores are commonly used in rectal cancer [37] and esophageal cancer [38].

Our research fits well into the current research landscape for defining new preoperative, predictive markers allowing better selection of patients with PM before major peritoneal surgery. For example, the preoperative PRGS after systemic therapy was predictive for OS in a cohort of 196 colorectal cancer patients treated with cytoreductive surgery and HIPEC for synchronous PM [8]. Similarly, PRGS after prior systemic chemotherapy was predictive for OS in a cohort of 47 patients with PM from gastrointestinal origin undergoing surgery or PIPAC. Taken together with our data, the evidence available strongly suggests that PRGS obtained from PM delivers crucial predictive information to distinguish between chemo-sensitive and-resistant tumors. This predictive information might be instrumental for improving therapeutic algorithms in patients with PM, in particular for selecting patients potentially benefitting from major peritoneal surgery [39].

The value of the PRGS for determining the response to chemotherapy was proven in a mouse model of PM from colorectal cancer. Previous clinical data evidenced the prognostic significance of PRGS in combination with peritoneal cytology [9]. PRGS was already used for objective therapeutic response in PM of colorectal [10, 11] gastric [13, 40] and various [41–43] origins. In our cohort of patients, repeated PRGS in the course of palliative chemotherapy had prognostic value. PRGS regression vs. progression was determined by comparing biopsies gathered just before the second vs. the first cycle of PIPAC, at a time interval of only six weeks. The PRGS improved in about two-thirds of patients, which is in line with previous reports [44]. The subgroup of responders to chemotherapy had a significantly better overall survival than those with a deteriorating mean PRGS. The short time between PIPAC is a methodological advantage enabling the identification of responders vs. non-responders at a very early point.

Peritoneal metastasis remains an unsolved challenge in modern oncology. Intratumoral heterogeneity is a fundamental property of cancer, in particular of PM. In the era of personalized cancer therapy, obtaining early prognostic and predictive information from tumor biopsies meets an urgent, unmet medical need. PRGS provides a solid methodological basis for reliable histological assessment of the therapeutic response in PM. However, further validation of PRGS in adequately powered studies is still needed before this score can be included in therapeutic algorithms and the WHO guidelines. A first step was the integration of PRGS into the international PIPAC registry to collect rapidly a large set of real-world data [45].

The next step will be to integrate meaningful molecular features that will strengthen PRGS’s prognostic and predictive accuracy and distinguish between subgroups of patients. For example, RAS/RAF mutations could be included since they impair survival after CRS and HIPEC in patients with PM of colorectal origin [46]. In gastric cancer, tumor heterogeneity was documented between the primary tumor and PM, between individual PM at different locations, and a 12-gene signature had prognostic significance [47]. In PM of ovarian origin also, changes in gene expression patterns during repeated intraperitoneal chemotherapy cycles were prognostic of overall survival [48]. Further development of PRGS, reflecting tumor biology, will stratify patients with PM based on long-term outcomes and improve patient selection for surgical, potentially curative therapy, vs. medical, palliative therapy. Moreover, profiling repeated PM biopsies under palliative chemotherapy (as PIPAC) gives new insights into the clonal evolution and the tumor escape mechanisms under palliative chemotherapy. This unique observational window offers new chances for unraveling tumor chemoresistance’s, molecular and immunological mechanisms in PM and beyond.
